# ALCAPA in an Octogenarian Woman: An Enigma

**DOI:** 10.14740/cr400w

**Published:** 2015-06-11

**Authors:** Santosh Kumar Sinha, Chandra Mohan Verma, Vinay Krishna, Ramesh Thakur, Barun Kumar, Amit Goel, Surendra Kumar, Ashutosh Kumar, Mukesh Jitendra Jha

**Affiliations:** a Department of Cardiology, LPS Institute of Cardiology, G.S.V.M. Medical College, G. T. Road, Kanpur, Uttar Pradesh, 208002, India

**Keywords:** ALCAPA syndrome, Arrythmia, Collateral circulation, Ischemic cardiomyopathy

## Abstract

ALCAPA syndrome (anomalous origin of the left coronary artery from the pulmonary artery) is an exceedingly rare disease but lethal with clinical expression from myocardial infarction, congestive heart failure to death during early infancy and rare survival to adulthood. A 75-year-old woman with ALCAPA syndrome presented with angina (Canadian Cardiovascular Society functional class II) over past 8 months. Physical examination was within normal limits except pan-systolic murmur at the apex. Electrocardiography displayed poor R progression in precordial leads and signs of left ventricular hypertrophy. Echocardiography established presence of continuous flow entering the pulmonary trunk and normal systolic function. Coronary angiogram showed absence of left coronary artery originating from aorta, dilated and tortuous right coronary artery (RCA) and abundant Rentrop grade 3 intercoronary collateral communicating with left coronary artery originating from pulmonary trunk which was also confirmed on CT angiogram thus establishing diagnosis of ALCAPA. Survival in eight decade with this anomaly is still an enigma.

## Introduction

ALCAPA (anomalous origin of the left coronary artery from the pulmonary artery) is a rare congenital cardiovascular anomaly. Because of profound disturbance of the normal physiology of the myocardium, survival to adulthood is rare [1, 2]. Patients with ALCAPA syndrome who survives beyond childhood often have symptoms varying from myocardial ischemia, syncope, arrhythmias to severe ischemic cardiomyopathy which of course will depend on the development of collateral circulation between right coronary artery (RCA) and left coronary system and up to 90% succumbs by third decade [2]. Survival beyond fourth or fifth decade with ALCAPA syndrome without surgical intervention is exceedingly rare. However, extensive collateral circulation may delay if not prevent ischemic damage as our patient had no past history of ischemic symptom. Sudden deaths occur in about 80-90% of the infants in absence of appropriate therapy as low pulmonary artery pressure results in coronary steal phenomenon, so survival beyond eighth decade is something very unusual.

## Case Report

**Figure 1:**
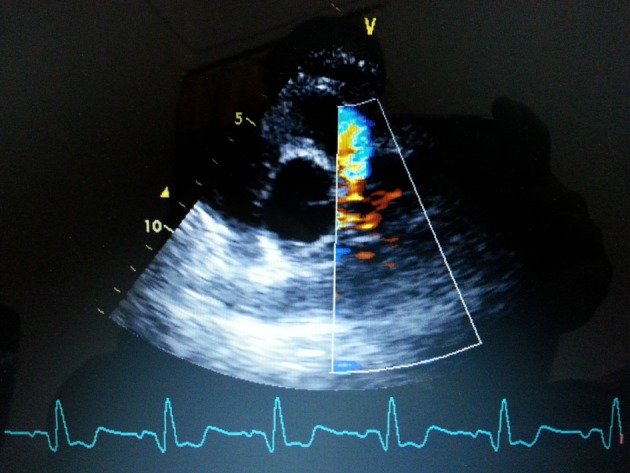
Transthoracic echo-parasternal short-axis view showing presence of continuous flow entering the pulmonary trunk.

**Figure 2:**
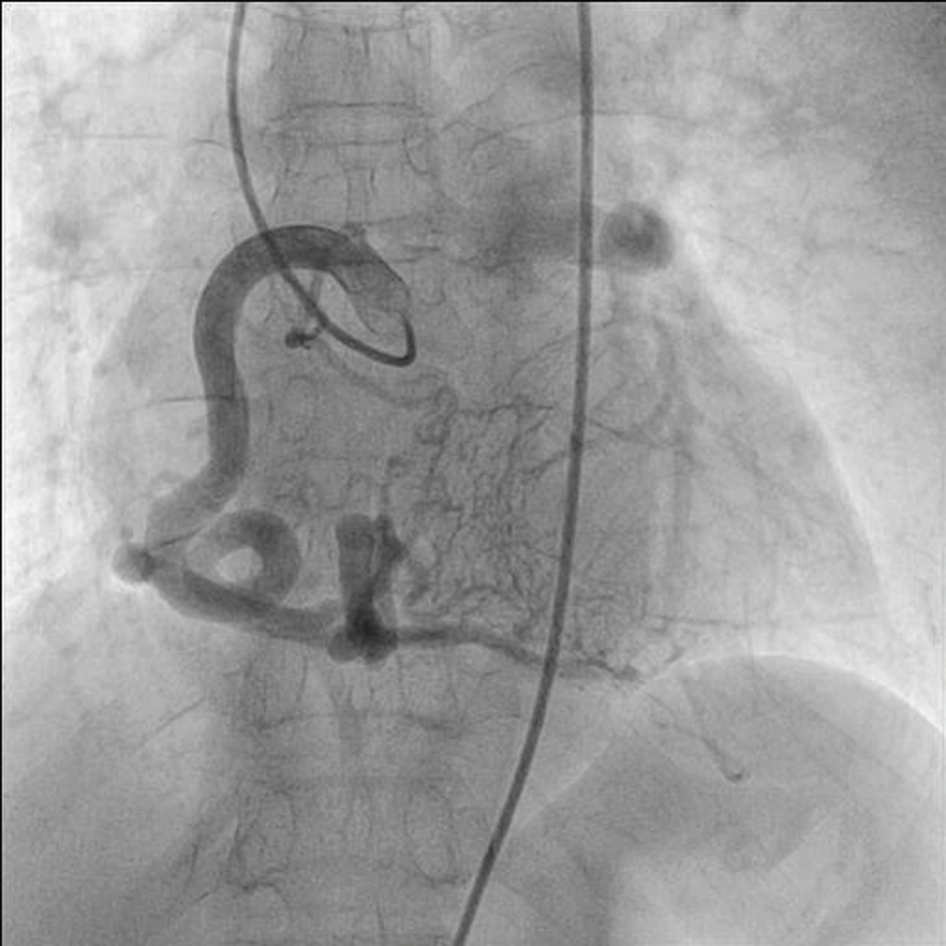
RAO (right anterior oblique) view of dilated and tortuous right coronary artery.

**Figure 3:**
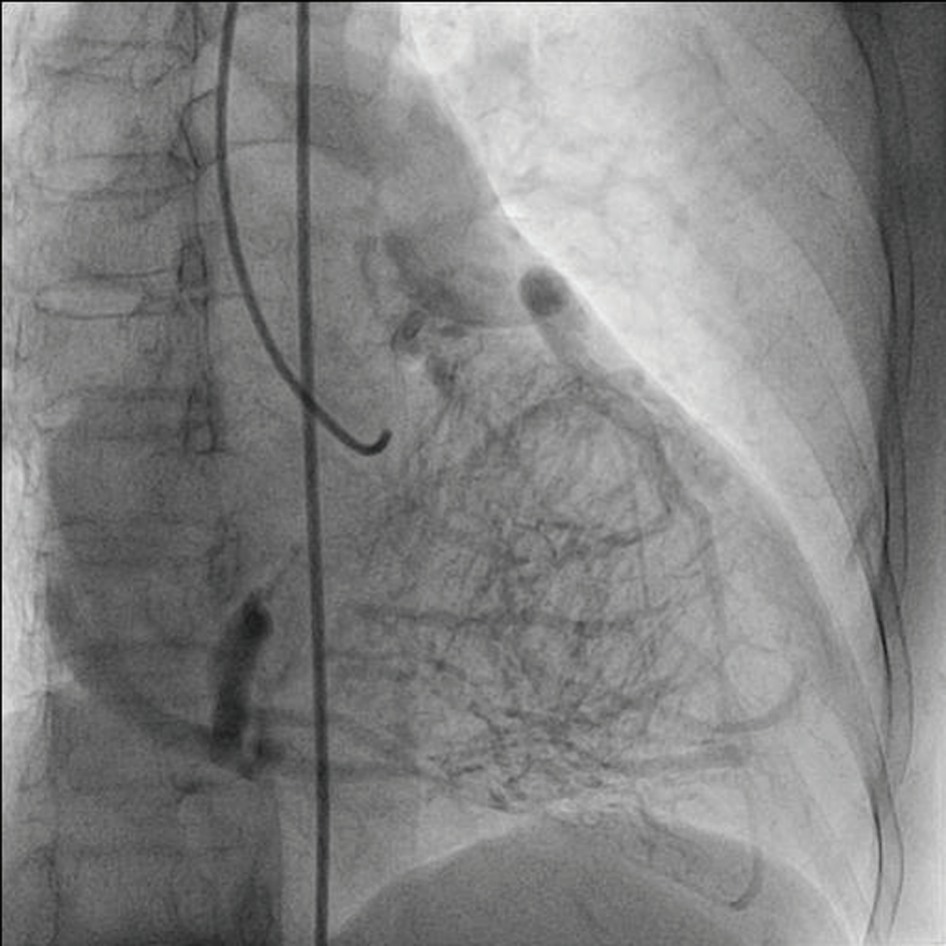
RAO (right anterior oblique) view of late phase showing abundant intercoronary anastomoses (Rentrop grade 3 intercoronary collateral) communicating with left coronary artery.

**Figure 4:**
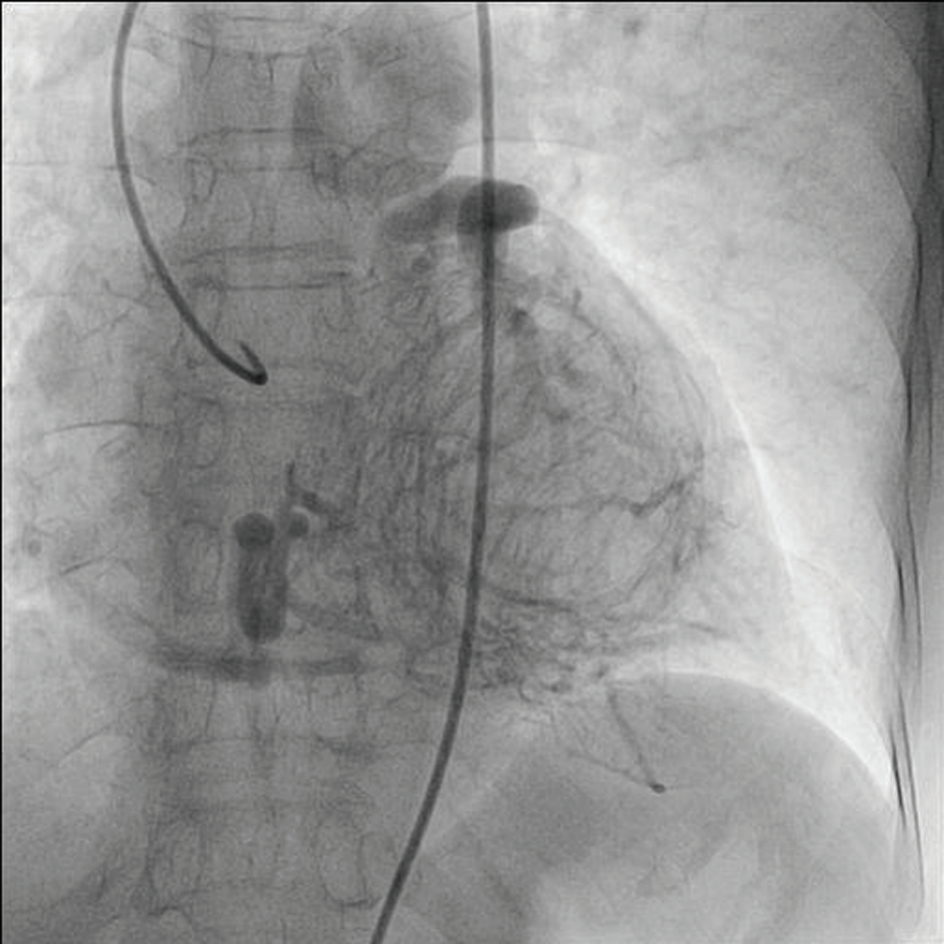
RAO (right anterior oblique) view of LAD and LCX arteries opacifying the LMCA and its origin from the main pulmonary artery.

**Figure 5:**
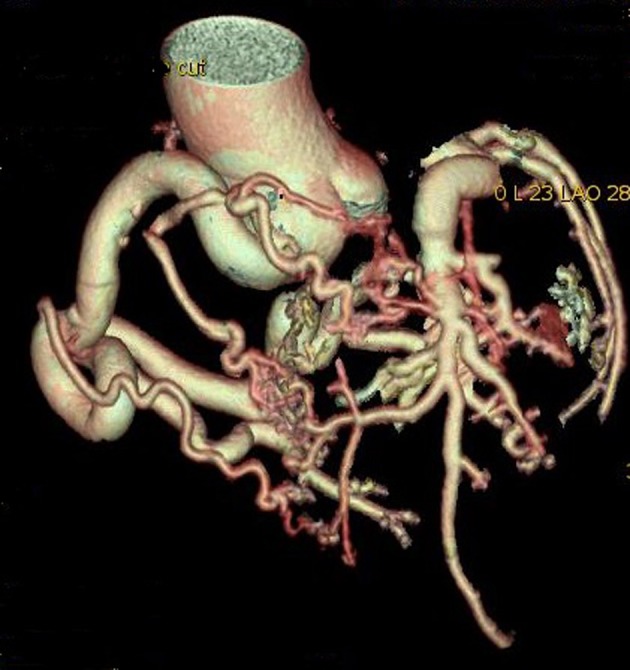
Reconstructed 3D CT angiogram showing dilated and tortuous right coronary artery with abundant intercoronary anastomoses communicating with left coronary artery and absence of its origin from aorta.

A 75-year-old woman presented with exertional angina that had progressed during previous 6 months to Canadian Cardiovascular Society functional class II at the time of admission to the hospital. Her pulse rate was 74 beat per minute and blood pressure was 152/94 mm Hg in right arm in supine position. There was no family history suggestive of coronary artery disease. Physical examination was unremarkable. On auscultation, pansystolic murmur (Levine 3/6) more prominent at the apex with radiation to axilla was heard. Mild cardiomegaly with enlargement of left ventricle and left atrium was noted on chest roentgenogram films. Electrocardiogram displayed poor R progression in precordial leads and ST-segment depression in leads V5-6, suggesting left ventricular hypertrophy. Trans-thoracic echocardiogram and color-Doppler established presence of continous flow entering the pulmonary trunk, mild mitral leak and normal systolic function (Fig. 1). Because of mild osteoarthritis of both knees, tread mill test could not be done. After proper consent, patient was taken to catheterization lab. Coronary cine-angiogram showed dilated and tortuous RCA with absence of a left coronary ostium in the left aortic sinus (Fig. 2). During the delayed phase, abundant intercoronary anastomoses (Rentrop grade 3 intercoronary collateral) were communicating with left coronary artery (Fig. 3) and still very delayed in filming sequence, retrograde flow from the left anterior descending and left circumflex coronary arteries was opacifying the left main coronary artery and its origin from the main pulmonary artery thus establishing diagnosis of ALCAPA (Fig. 4). Similar finding was recognized on reconstructed 3D coronary CT angiogram thus confirming ALCAPA (Fig. 5). Though surgical treatment by recreating a dual coronary perfusion is usually warranted regardless of the symptoms or myocardial function, it was decided not to intervene surgically as risk of cardiac surgery was outweighing the benefit. She was discharged in stable condition with medical management.

## Discussion

The estimated incidence of ALCAPA is 1/300,000 live births (between 0.24% and 0.46% of all congenital cardiac anomalies) [3]. Wesselhoft et al [4] have proposed the clinical spectrum of ALCAPA spanning from infantile syndrome, mitral regurgitation, syndrome of continuous murmur and sudden death in adulthood. Most of the adults are asymptomatic, but some may experience angina on exertion, cardiac arrhythmias and sudden death. Patient survival without surgical intervention depends on development of unusually abundant collateral channels from the RCA and closure of the ductus arteriosus. ALCAPA develops before birth and as the systemic and pulmonary arterial pressures are equal, there is anterograde flow in both the left and the right coronary. In the neonatal period as the pulmonary artery pressure falls and ductus arteriosus closes, the flow in the left coronary artery reverses. The more collateral channels exist between RCA and left coronary system, the longer the patient may survive. Another contributing factor may be the area supplied by the ALCA. The smaller the area supplied by the ALCA, the less extensive the myocardial ischemia is likely to be. In few adult cases, RCA supplies not only the posterior wall of the left ventricle but also its lateral wall. May be this reduction in the area of the myocardium supplied by the ALCA would favor survival [4]. However, even extensive collateral circulation may not totally prevent ischemic damage though can delay it as low pulmonary artery pressure may still result in coronary steal phenomenon, which may cause sudden deaths. Nearly 80-90% of the infants succumb in absence of appropriate therapy. Survival past fourth or fifth decade is rare and untreated ALCAPA in the elderly is exceedingly rare [5]. We believe RCA may have supernormal flow reserve to account for her survival but it will still remain an enigma.
